# The Endonasal Endoscopic Transsphenoidal Approach to Paediatric Optic Chiasma Glioma: A Case Report and Literature Review

**DOI:** 10.7759/cureus.52649

**Published:** 2024-01-21

**Authors:** Norah S Al Shareef, Ali Almomen, Retaj Alawadhi, Abdulrhman Alkhatib, Sultan Alsaiari

**Affiliations:** 1 Medicine and Surgery, Royal College of Surgeons in Ireland, Dublin, IRL; 2 Rhinology and Skull Base Surgery, King Fahad Specialist Hospital, Dammam, SAU; 3 Otolaryngology, King Fahad Specialist Hospital, Dammam, SAU; 4 Neurosurgery, King Fahad Specialist Hospital, Dammam, SAU

**Keywords:** neurofibromatosis type, otolaryngology, endoscopic endonasal skull base surgery, endonasal endoscopic transsphenoidal approach, optic pathway gliomas

## Abstract

Optic pathway gliomas (OPGs) are rare pediatric tumors that pose significant challenges in management due to their location and clinical manifestations. Traditional transcranial approaches have been the mainstay for surgical intervention, but recent advancements in endoscopic endonasal transsphenoidal surgery offer a less invasive alternative. Here, we present a case of a 10-year-old female child with neurofibromatosis type-1 and an aggressive OPG who underwent endoscopic endonasal transsphenoidal debulking surgery. The pre-operative evaluation confirmed complete vision loss, and imaging revealed tumor progression. The surgery was successful, resulting in a subtotal resection and a diagnosis of pilocytic astrocytoma, WHO grade 1. Post-operative assessments showed no complications. This case highlights the feasibility of the endoscopic endonasal transsphenoidal approach for OPGs and emphasizes the importance of careful patient selection and multidisciplinary collaboration in achieving successful outcomes for these challenging tumors.

## Introduction

Optic pathway gliomas (OPGs), also known as visual pathway gliomas, are debilitating tumors that account for 3-5% of all pediatric brain tumors. They are most commonly WHO grade 1 pilocytic astrocytomas and frequently occur in patients with neurofibromatosis type 1. The location of these tumours can result in partial or complete visual impairment, endocrine dysfunction, and hydrocephalus. Their proximity to other critical brain structures, the histologically benign nature, and the considerable risk of complications following surgical intervention make OPGs a challenging entity to manage [1,2]. In cases where surgery is considered, these lesions are usually approached by transcranial routes. The use of the endoscopic endonasal transsphenoidal approach (EETA) for optic glioma as an alternative to the traditional transcranial approach is relatively new, and there is limited literature on its use. We report a case of a pediatric optic pathway glioma with an early onset and an aggressive clinical course that underwent EETA for subtotal resection and biopsy.

## Case presentation

A 10-year-old female child, with neurofibromatosis type-1 since birth, was diagnosed with an extensive optic pathway glioma and experienced complete vision loss at three years of age. She underwent three lines of chemotherapy from 2015 to 2019. Initially treated with carboplatin and vincristine, she developed an allergy and was shifted to weekly vinblastine in September 2016. Vinblastine continued for 53 weeks until October 2017. Two years after discontinuation of chemotherapy, the patient exhibited disease progression on imaging. A third line of chemotherapy, irinotecan/bevacizumab, was initiated and continued for three cycles but was discontinued in 2019 due to further disease progression on imaging. Subtotal resection and biopsy were warranted to rule out malignancy transformation of the progression.

**Figure 1:**
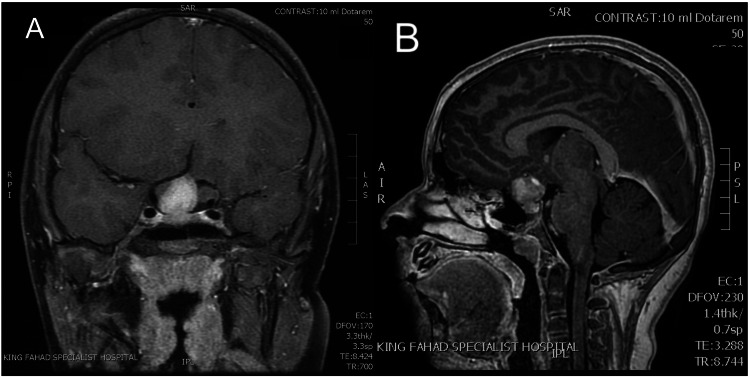
(A) Coronal (B) Sagittal T1-weighted contrast-enhanced MRI

The pre-operative assessment included ophthalmic, endocrinology, and brain imaging evaluations. Ophthalmic evaluation confirmed complete vision loss with no light perception in assessing visual acuity. Extraocular movements (EOM) and corneal reflex were intact, and fundoscopy showed bilateral optic nerve atrophy. The pediatric endocrinology assessment was unremarkable. Brain MRI in coronal and sagittal planes revealed the right chiasmatic part of the optic glioma (Figure 1).

**Figure 2:**
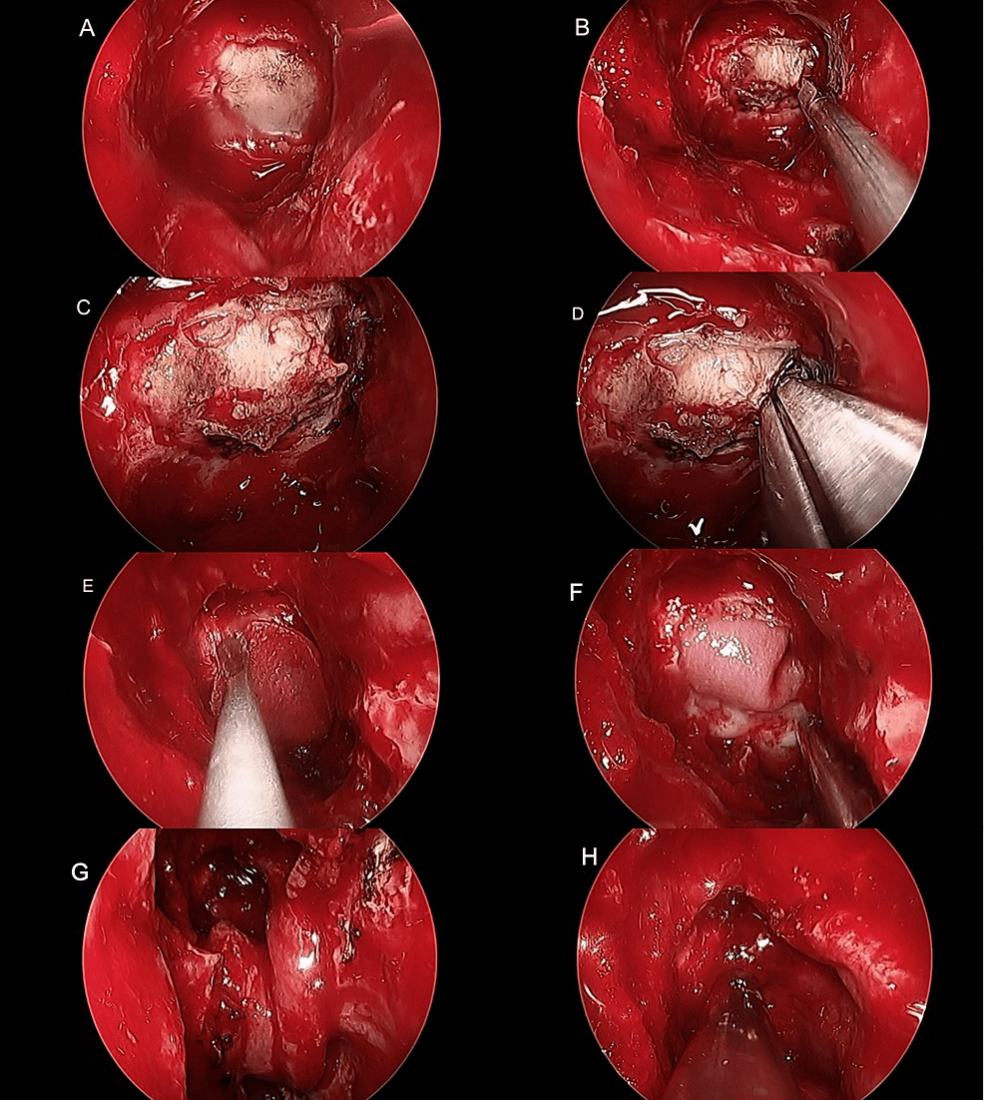
(A) Transsphenoidal full exposure of tumor with intact dura; (B) Dural incision; (C) Full exposure of tumor; (D) Transsphenoidal debulking of tumor; (E) Dural reconstruction with free dural graft; (F) The dural graft secured underlay covering the tumor; (G) Pedicled nasoseptal flap for skull base reconstruction; (H) Gelfoam on the final reconstructed skull base

The patient was referred to the neurosurgery and rhinology skull base team and underwent endoscopic endonasal transsphenoidal debulking surgery on March 16, 2021. The tumor was fully exposed through a wide endonasal endoscopic transsphenoidal approach, the dura was incised, debulking of the tumor was achieved, and the skull base defect was reconstructed using a free dural underlay graft and a pedicelled nasoseptal flap for the final skull base reconstruction (Figure 2). The operation was successful with no intraoperative complications. Histopathology reported pilocytic astrocytoma, WHO grade 1.

The patient was admitted to the pediatric intensive care unit (PICU) for two days and then transferred to the pediatric general ward. The post-operative assessment revealed no evidence of CSF leak, pneumocephalus, meningitis, acute intraparenchymal, or subarachnoid hemorrhage. Additionally, ophthalmic and endocrinology assessments showed the same baseline findings. The patient showed a smooth recovery without complications and was discharged on March 24, 2021. The two-week post-operative follow-up showed good recovery with no active complaints; however, the patient was lost to follow-up due to poor compliance. 

## Discussion

Optic chiasma gliomas are rare tumors that can present unique challenges in surgical intervention, particularly in the pediatric population. Traditionally, these tumors have been approached through open craniotomies, but recent advancements in endonasal endoscopic transsphenoidal surgery provide a less invasive option. This case report illustrates the successful application of the EETA in treating a pediatric patient with an optic chiasma glioma.

Several advantages are associated with this minimally invasive technique, including reduced surgical trauma, shorter hospital stays, and improved cosmetic outcomes. Avoiding a craniotomy also minimizes the risk of damage to adjacent critical structures, reducing the potential for postoperative complications [1]. Furthermore, the endoscopic approach offers a direct, magnified view of the tumor and surrounding anatomy, enhancing the surgeon's ability to achieve gross total resection while preserving vital structures like the pituitary gland. The absence of external incisions also lowers the risk of CSF leaks and infections [2,3]. However, it is important to acknowledge that this approach may not be suitable for all cases, and patient selection should be carefully considered. Factors such as tumor size, location, and the surgeon's experience with endoscopic techniques should be taken into account. The EETA is a viable and effective surgical option for pediatric patients with optic chiasma gliomas.

The available literature contains limited information and experiences regarding the use of endoscopic approaches for OPGs situated in the chiasma-hypothalamic region [4]. Published cases typically consist of small case series or mixed case series that explore endoscopic endonasal approaches for suprasellar region tumors. Our case specifically focuses on OPGs located in the chiasma-hypothalamic region and treated using the EETA. While there is no consensus regarding the optimal treatment for OPGs, observation is primarily favored, especially for asymptomatic patients. In cases where patients experience symptoms, there remains ongoing debate between using adjuvant treatments and opting for surgical intervention as the initial treatment approach.

Certain authors have outlined criteria for surgical intervention in OPG patients, typically considering surgery when there is single nerve involvement leading to progressive proptosis or vision loss, exophytic chiasmatic tumors causing mass effect, or inducing hydrocephalus. Hill et al., in their recent article, emphasized the importance of surgical expertise in determining the surgical indications for this rare disease [5]. They recommended surgery in cases where the tumor is located in the chiasma-hypothalamic region and results in visual loss or disrupts CSF circulation due to mass effect, leading to obstructive hydrocephalus. In instances where symptoms are clinically tolerable, they advocated postponing surgery and opting for conservative treatment as the initial approach. However, they suggested surgery for patients whose symptoms worsen or show radiological progression despite multiple chemotherapy or biologic treatment attempts. Goodden et al. stressed the significant role of surgery in diagnosing OPGs, controlling tumor growth, and alleviating mass effects. They asserted that primary surgical removal of the tumor without additional treatments is a safe and effective approach [4].

The 2011 guide published by the Society of British Neuro-Oncology also mentioned surgery as a potential option for patients with vision loss on one side to preserve their remaining vision and potentially manage hydrocephalus. EETA to the skull base has been extensively explained in the context of more common skull base tumors like pituitary adenomas, meningiomas, and craniopharyngiomas. While the effectiveness of EETA for tumors located outside the brain tissue within the skull base is well-established, its role in dealing with tumors within the brain tissue itself is not as clear. There are only a few reports on this topic in the literature, and most of these reports are either single-case accounts or involve small groups of cases. Some examples of reports on the use of EETA for removing brain tissue tumors include cases of pontine ependymoma, pontine cavernoma, hypothalamic glioma, and germ-cell tumors. There is limited prior information on the use of EETA for OPGs [5], and as far as we know, there are no reports discussing its utility in pediatric OPGs. Most of the available cases are part of larger mixed case series that discuss the use of EETA for various lesions located above the sellar region. Zoli et al. reported their specific experience with EETA for OPGs [6]. They presented five cases with an average age of 32 years (ranging from 13 to 44 years) [6]. The most common clinical symptoms in these cases included visual impairments and abnormalities in the hypothalamic-pituitary axis. Four of these cases were diagnosed as pilocytic astrocytomas, while the fifth case was identified as a pilomyxoid astrocytoma [6].

This case report demonstrates the success of EETA in achieving tumor resection with minimal complications, leading to significant improvements in visual function and quality of life for the patient. As with any surgical approach, a multidisciplinary team and careful patient selection are essential to ensure optimal outcomes.

## Conclusions

The presented case highlights the successful application of the EETA as a minimally invasive surgical option for the management of pediatric OPGs. OPGs, particularly in the chiasma-hypothalamic region, present unique challenges, and the choice between surgery and conservative management remains a topic of debate. In this case, EETA allowed for tumor debulking with minimal surgical trauma and favorable cosmetic outcomes. Factors such as tumor size, location, and the surgeon's experience with endoscopic skull base techniques should guide patient selection. This case report contributes to the limited literature on the use of EETA for pediatric OPGs, specifically those situated in the chiasma-hypothalamic region. It emphasizes the importance of a multidisciplinary approach and the need for ongoing research to refine surgical indications and optimize outcomes for patients with these challenging tumors.
